# Supplementary material for: The Role of the Second Extracellular Loop of Norepinephrine Transporter, Neurotrophin-3 and Tropomyosin Receptor Kinase C in T Cells: A Peripheral Biomarker in the Etiology of Schizophrenia
Source: Int J Mol Sci. 2021 Aug 7;22(16):8499. doi: 10.3390/ijms22168499 (PMC8395201; doi:10.3390/ijms22168499)

# Supplementary material

**Supplementary Table 1:** Main antipsychotic treatment.

| Current treatment | Number of patients (N) | % of patients | Dose (mg)* |
|---|---|---|---|
| **First-episode psychosis (FEP)** | | | |
| **Drug näive** | 12 | 22.22 | - |
| **Chronic phase** | | | |
| **Aripiprazole** | 8 | 14.82 | 19.43 ± 8.42 |
| **Aripiprazole + Olanzapine** | 1 | 3.70 | 20 + 20 |
| **Aripiprazole + Olanzapine + Zuclopenthixol** | 1 | 1.85 | 15 + 20 + 200 |
| **Aripiprazole + Clozapine** | 1 | 1.85 | 415 + 25 |
| **Aripiprazole + Quetiapine** | 1 | 1.85 | 10 + 50 |
| **Paliperidone** | 2 | 1.85 | 206.05 ± 56.50 |
| **Olanzapine** | 8 | 14.81 | 13.75 ± 4.15 |
| **Olanzapine + Risperidone** | 1 | 1.85 | 15 + 2 |
| **Risperidone** | 7 | 12.96 | 5.38 ± 2.06 |
| **Risperidone + Olanzapine + Aripiprazole** | 1 | 1.85 | 20 + 6 + 400 |
| **Ziprasidone + Zuclopenthixol** | 1 | 1.85 | 40 + 200 |
| **Clomethiazole** | 1 | 1.85 | 192 |
| **Clozapine** | 9 | 16.68 | 322.22 ± 103.04 |

Antipsychotic treatment of patients diagnosed with schizophrenia. * Dose or mean ± SD of dose.

**Supplementary Table 2:** Correlation between NT-3, TrkC and $NET_{ext}$ levels and clinical variables.

| PARAMETERS | NT-3 | | $NET_{ext}$ | | TrkC | |
|---|---|---|---|---|---|---|
| | **r** | ***p*** | **r** | ***p*** | **r** | ***p*** |
| **PANSS Positive** | -0.1384 | 0.4502 | 0.1566 | 0.3840 | -0.2959 | 0.1603 |
| **PANSS Negative** | -0.0023 | 0.9901 | 0.1113 | 0.5312 | 0.1250 | 0.5606 |
| **PANSS General** | -0.1348 | 0.4620 | 0.1283 | 0.4769 | -0.2822 | 0.1815 |
| **PANSS Total** | -0.1229 | 0.5029 | 0.1756 | 0.3282 | -0.1771 | 0.4077 |
| **Illness onset** | -0.2861 | 0.1124 | -0.0728 | 0.6873 | -0.1154 | 0.5912 |
| **Duration of illness** | -0.0705 | 0.7013 | 0.0418 | 0.8172 | 0.0514 | 0.8114 |
| **NLR** | 0.1382 | 0.4507 | -0.1177 | 0.5147 | 0.0303 | 0.8882 |

Statistically significant according to Pearson's correlation coefficients: $P \leq 0.05$* (two-tailed). PANSS: Positive and Negative Syndrome Scale. NLR: neutrophil-lymphocyte ratio.

**Supplementary Figure 1:** CABS-dock workflow.

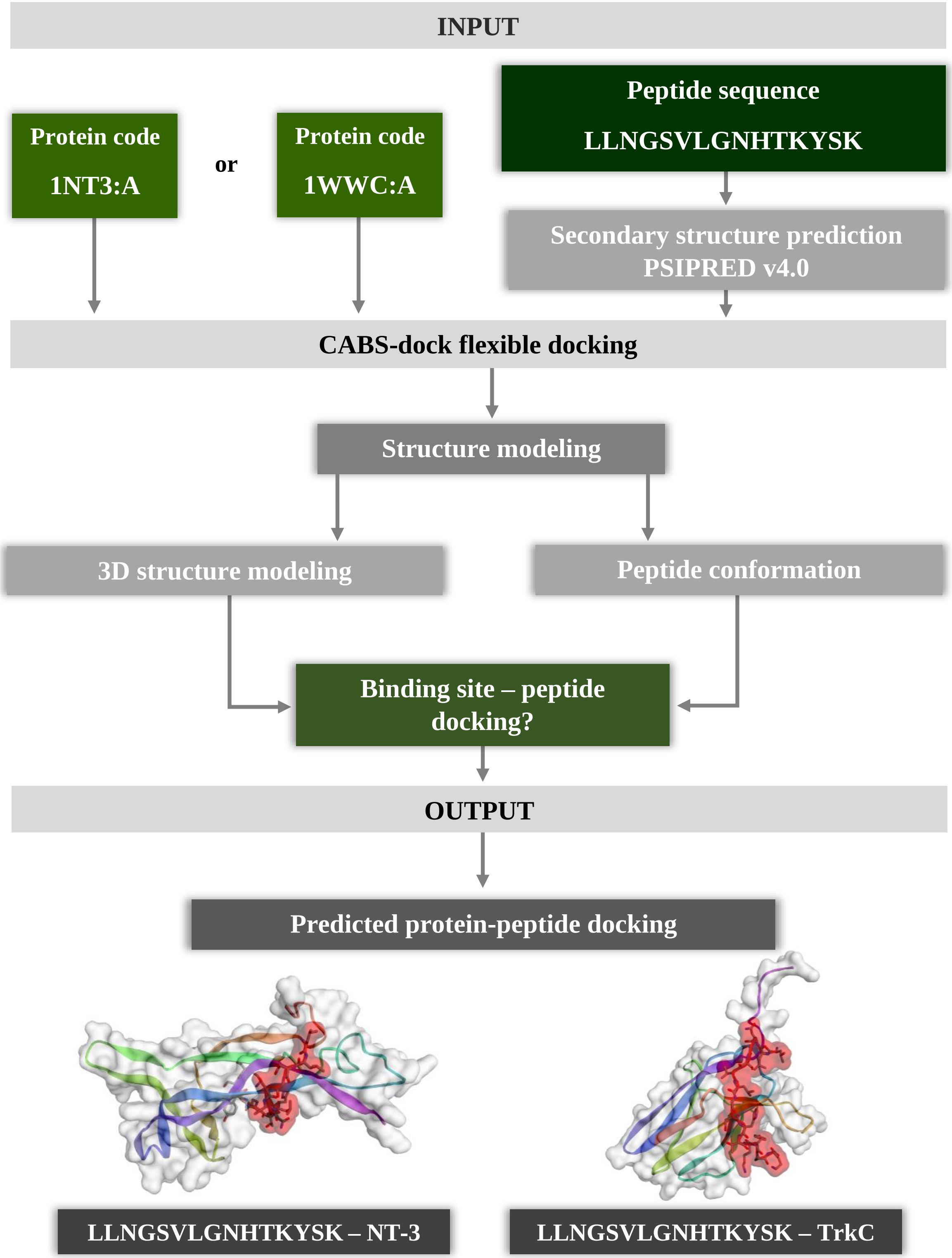


**Legend:** CABS-dock web server workflow used to predict binding models of protein-peptide

**Supplementary Figure 2:** Fluorescence microscopy images of blood lymphocytes.

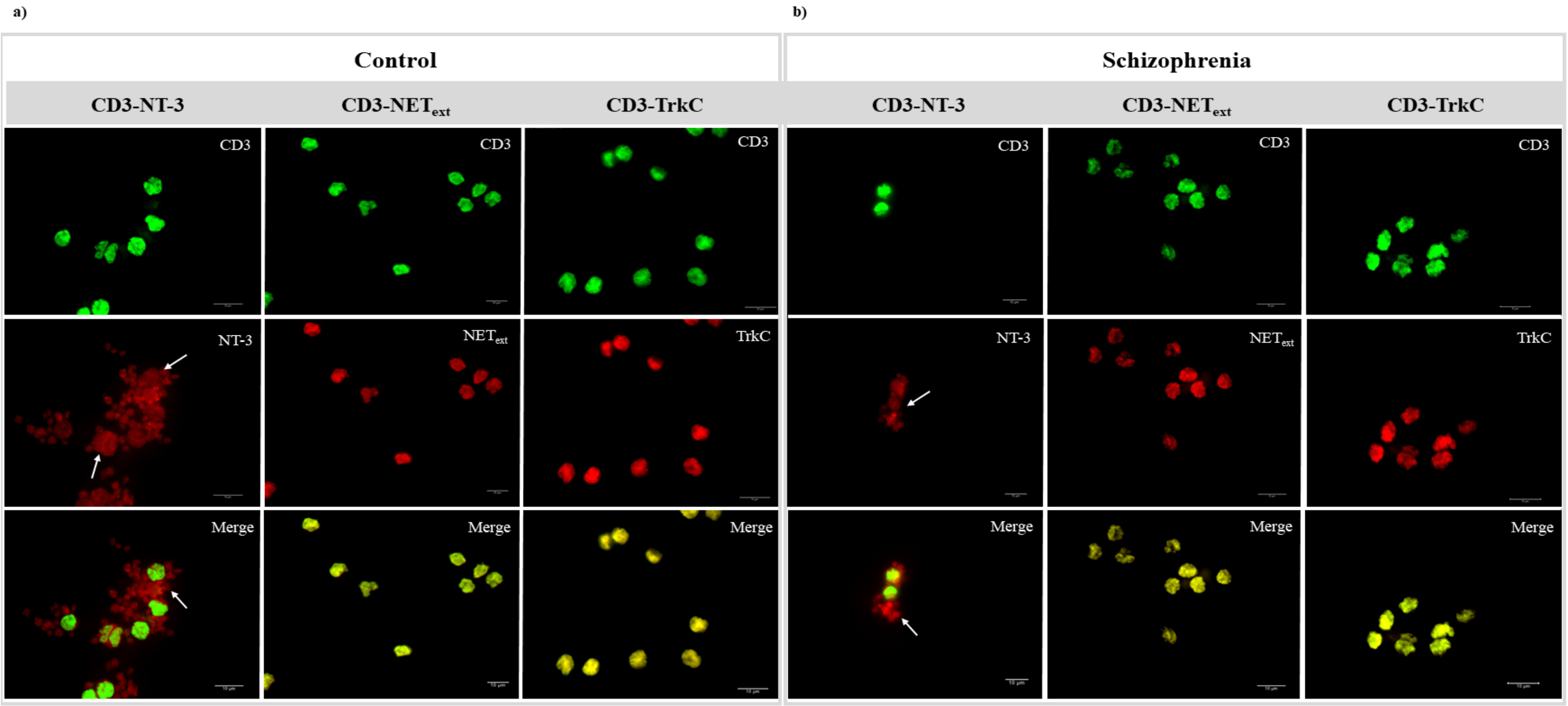


**Legend:** Lymphocytes stained with anti-CD3 + anti-Alexa Fluor 488 (green color) and anti-NT-3 + anti-Alexa Fluor 594 (red color) antibodies expanded from peripheral blood mononuclear cells with an HCX PL APO CS 63.0x1.40 OIL UV objective. a) Immunostaining of lymphocytes of a control that showed the expression of CD3 (green), NT-3, $NET_{ext}$ and TrkC (red). b) Immunostaining of lymphocytes of a patient with schizophrenia that showed the expression of CD3 (green), NT-3, $NET_{ext}$ and TrkC (red). The white arrows indicate the differential expression of NT-3 in the control compared to the patients with schizophrenia.

**Supplementary figure 3:** Top ten of predictive protein-peptide docking for NT-3.

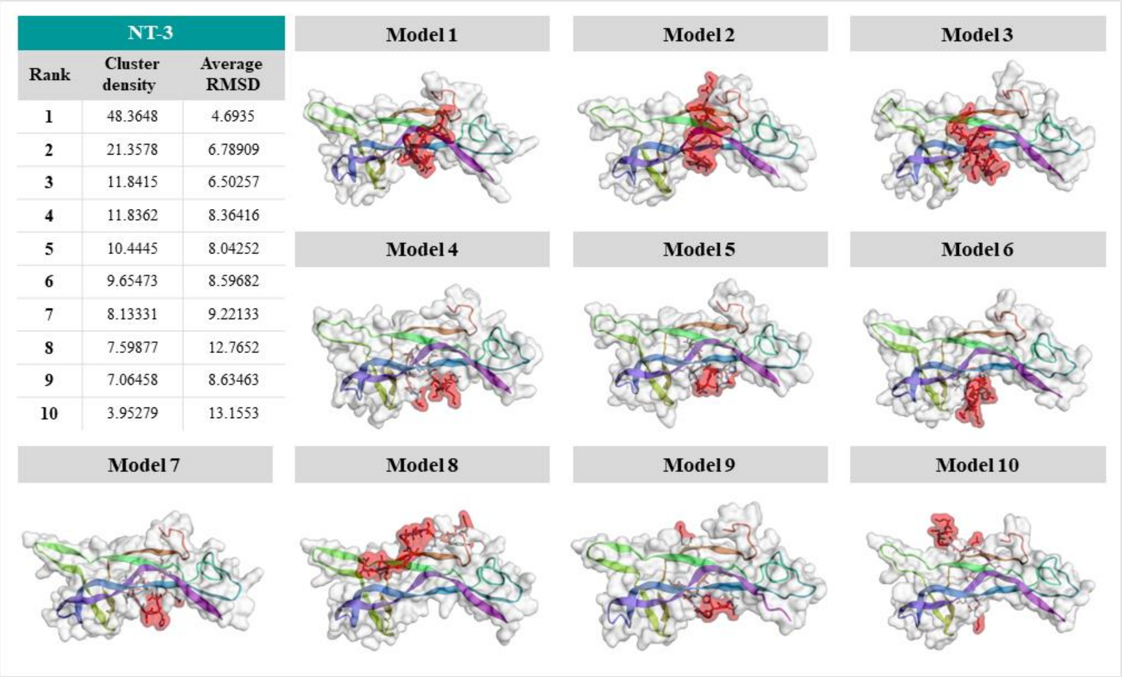


| NT-3 | | |
|---|---|---|
| Rank | Cluster density | Average RMSD |
| 1 | 48.3648 | 4.6935 |
| 2 | 21.3578 | 6.78909 |
| 3 | 11.8415 | 6.50257 |
| 4 | 11.8362 | 8.36416 |
| 5 | 10.4445 | 8.04252 |
| 6 | 9.65473 | 8.59682 |
| 7 | 8.13331 | 9.22133 |
| 8 | 7.59877 | 12.7652 |
| 9 | 7.06458 | 8.63463 |
| 10 | 3.95279 | 13.1553 |

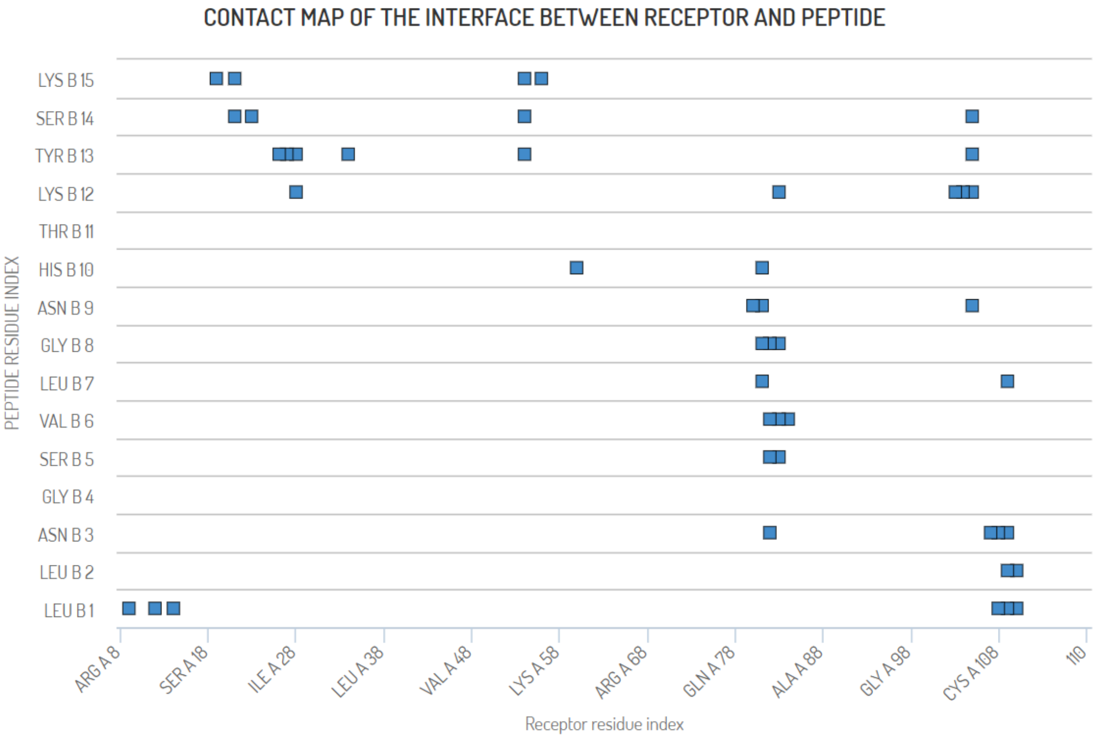


**Legend:** Computational modelling of protein-peptide docking of NT-3 (1nt3:A) and the peptide (NEText: LLNGSVLGNHTKYSK). Clustering details of the ten models are shown in the respective tables (cluster density is the number of elements in cluster (N)/Average of RMSD). The contact map shows the pairs of peptide/NT-3 residues closer than 4.5 Å in the complex.

**Supplementary figure 4:** Top ten of predictive protein-peptide docking for TrkC.

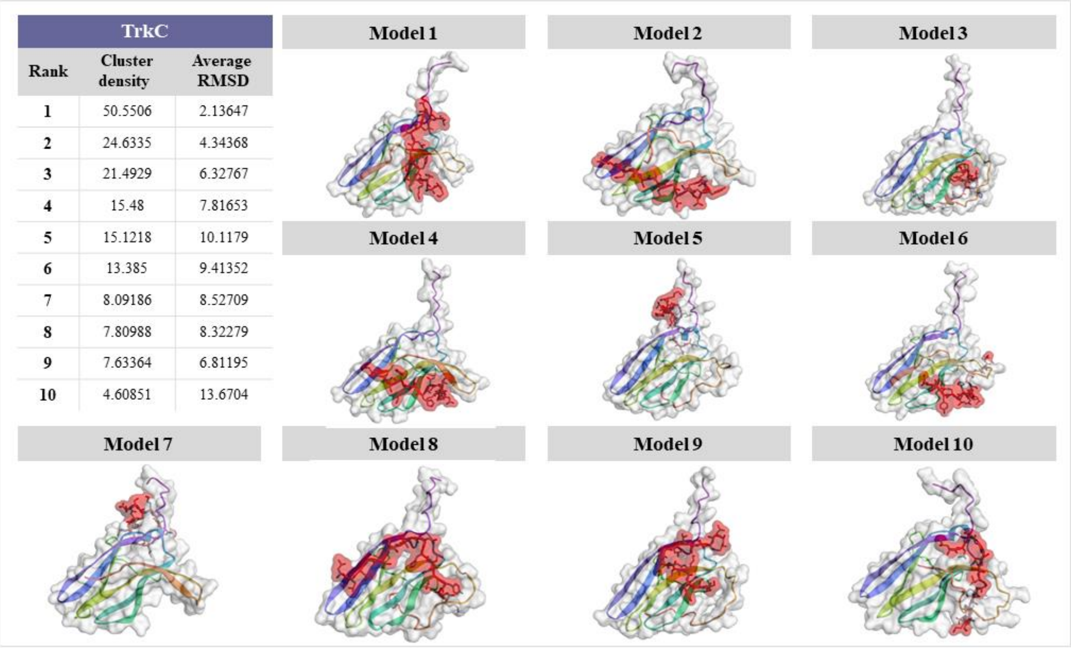


| TrkC | | |
|---|---|---|
| Rank | Cluster density | Average RMSD |
| 1 | 50.5506 | 2.13647 |
| 2 | 24.6335 | 4.34368 |
| 3 | 21.4929 | 6.32767 |
| 4 | 15.48 | 7.81653 |
| 5 | 15.1218 | 10.1179 |
| 6 | 13.385 | 9.41352 |
| 7 | 8.09186 | 8.52709 |
| 8 | 7.80988 | 8.32279 |
| 9 | 7.63364 | 6.81195 |
| 10 | 4.60851 | 13.6704 |

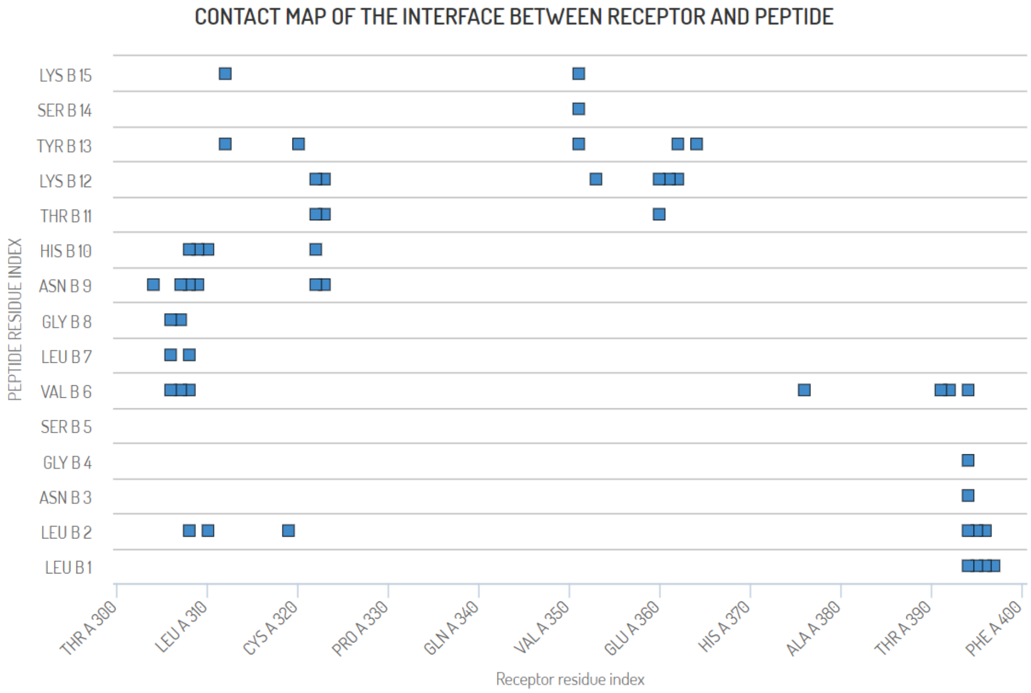


**Legend:** Computational modelling of protein-peptide docking of TrkC (1wwc:A) and the peptide (NEText: LLNGSVLGNHTKYSK). Clustering details of the ten models are shown in the respective tables (cluster density is the number of elements in cluster (N)/Average of RMSD). The contact map shows the pairs of peptide/TrkC residues closer than 4.5 Å in the complex.

**Original immunofluorescence images.**

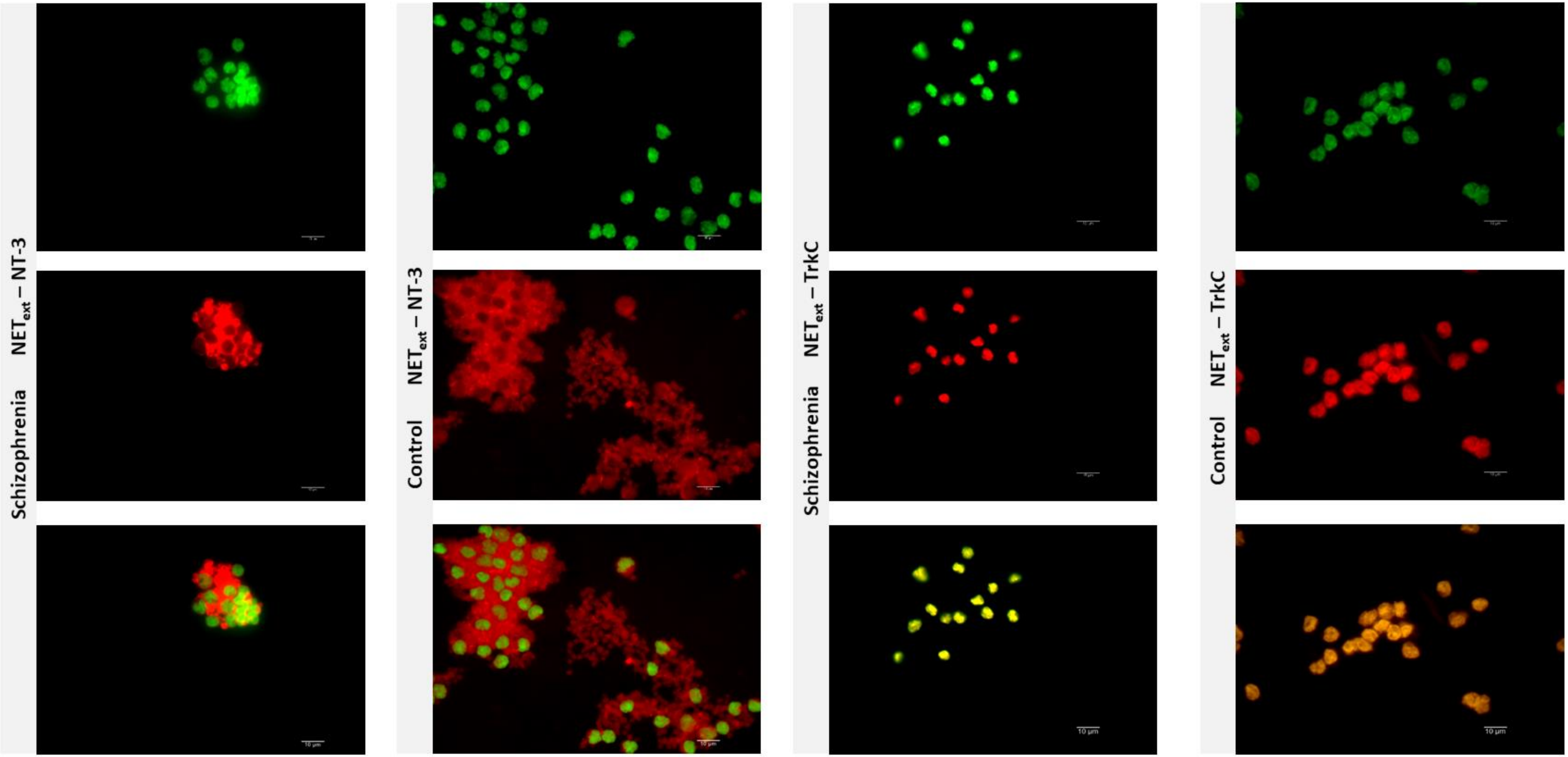

**Original Western blots:**

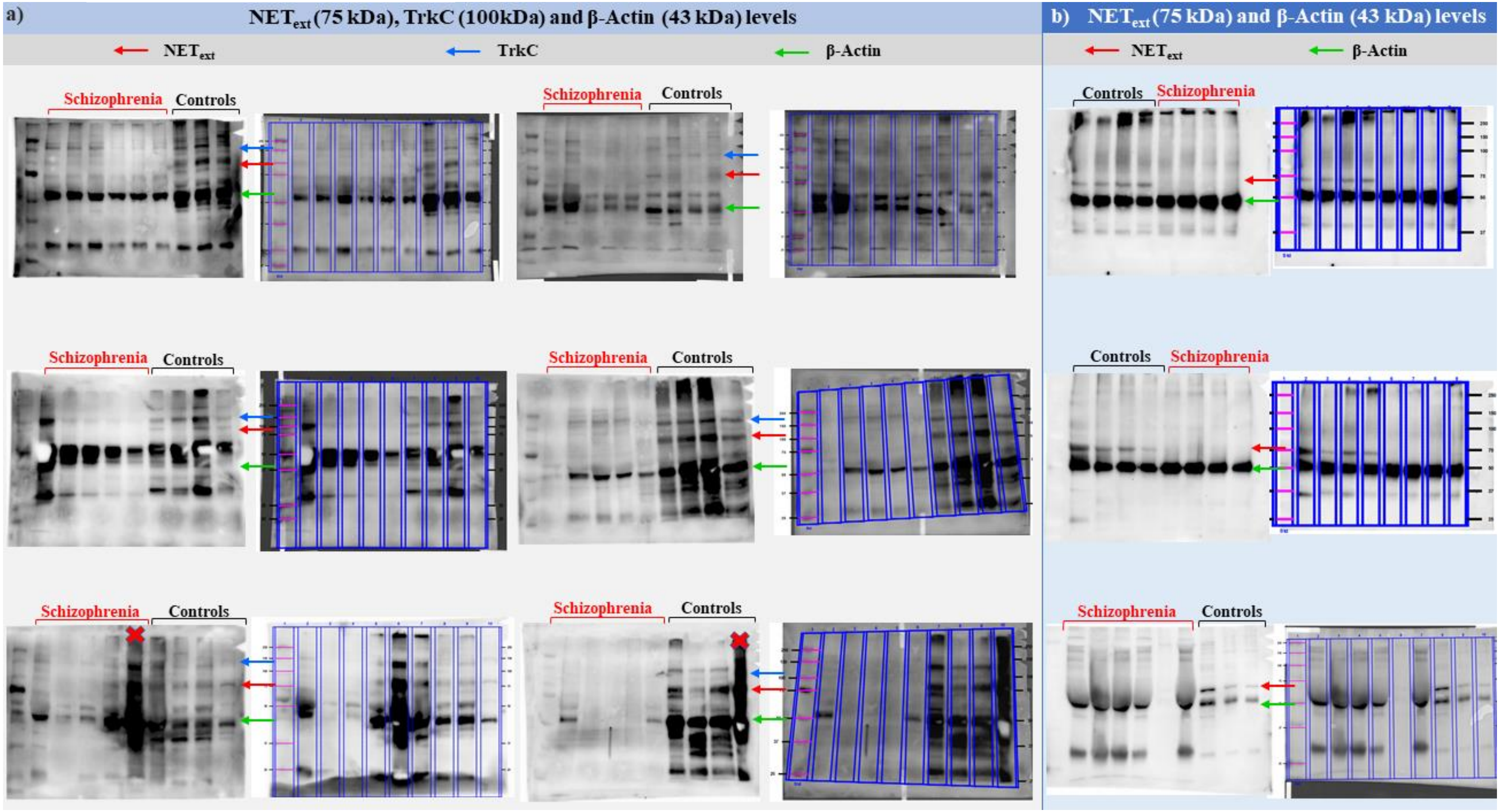


a) Simultaneous detection of NET$_{ext}$, TrkC and β-Actin using T cell samples. b) Original blots of NET$_{ext}$ and β-Actin using T cell samples. Blue arrows correspond to TrkC (≈100 kDa), red arrows correspond to NET$_{ext}$ (75 kDa and green arrows correspond to β-Actin (43 kDa).

**Original Co-IPs:**

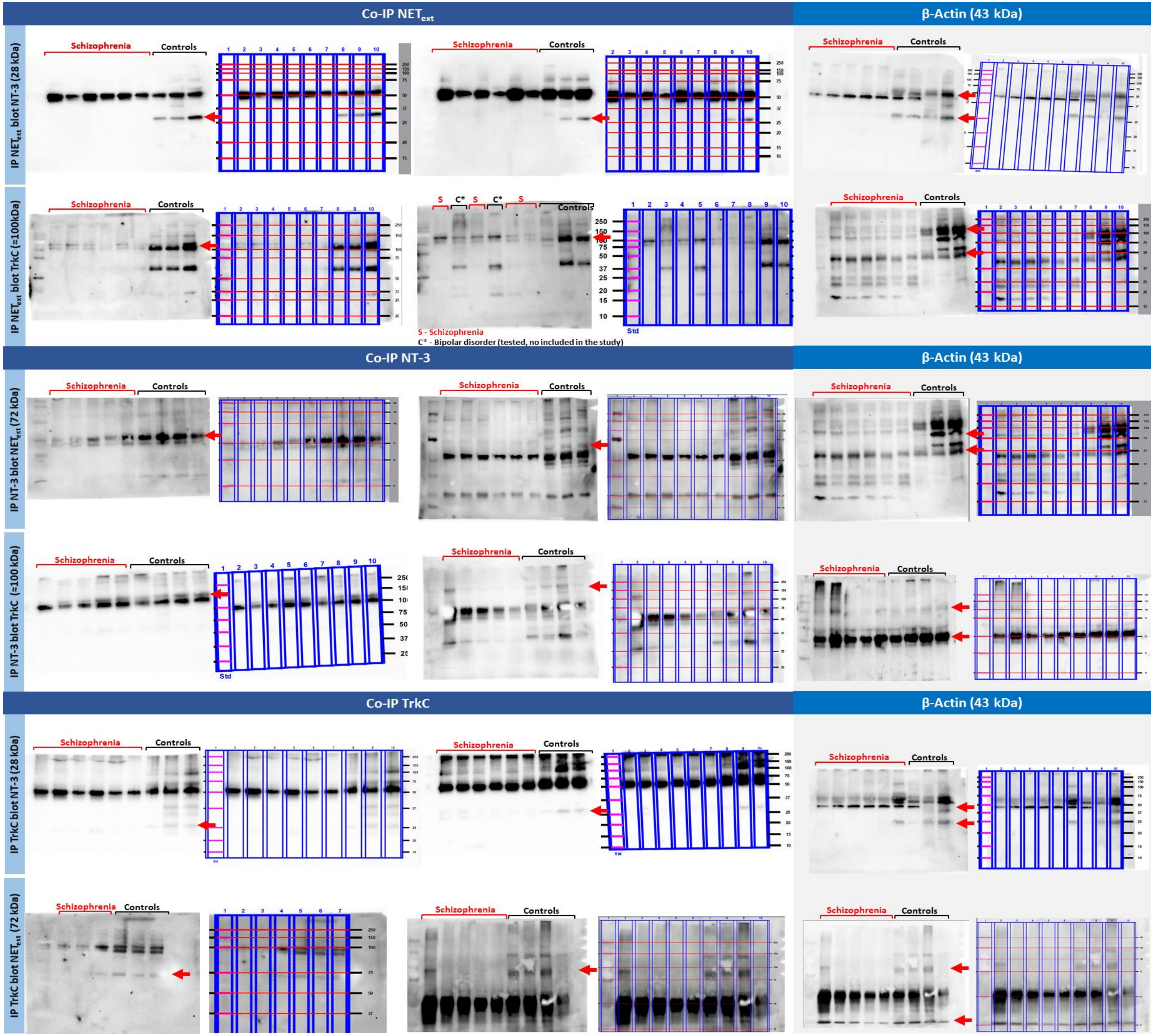

Supplement: Supplementary file 1 [file ijms-22-08499-s001.zip › ijms-1271061-SI.pdf]
